# Supplementary material for: Differences in COVID-19 testing perceptions among caregivers of children with medical complexity by rurality
Source: PLoS One. 2025 May 13;20(5):e0323651. doi: 10.1371/journal.pone.0323651 (PMC12074607; doi:10.1371/journal.pone.0323651)
Supplement: S1 Table — (DOCX) [file pone.0323651.s001.docx]

**Supporting information Table 1. Caregiver and Child Demographic Characteristics by Response Status, *n* = 3080.**

|  | *n respondents  (column %)* | *n non-respondents (column %)* | *p-value* |
| --- | --- | --- | --- |
| ***Caregiver Characteristics*** | | | |
| **Caregiver Race/Ethnicity Binary** | | | |
| White, Non- Hispanic | 1162 (81.2) | 1197 (72.6) | <.0001 |
| All Other Races & Ethnicities | 270 (18.8) | 451 (27.4) |  |
| **Primary Payor Group** | | | |
| Public | 278 (19.4) | 473 (28.7) | <.0001 |
| Private | 1147 (80.1) | 1149 (69.7) |  |
| Other | 7 (0.5) | 26 (1.6) |  |
| **Language** | | | |
| English | 1396 (97) | 1556 (94) | <.0001 |
| Not English | 36 (3) | 92 (6) |  |
| **Rurality** | | | |
| Rural | 359 (25) | 428 (26) | 0.57 |
| Urban | 1073 (75) | 1220 (74) |  |
| ***Child Characteristics*** | | | |
| **Sex** | | | |
| Male | 757 (52.9) | 852 (51.7) | 0.52 |
| Female | 675 (47.1) | 796 (48.3) |  |
| **Age** | | | |
| 5 – 10 Years old | 339 (30.3) | 361 (28.5) | 0.65 |
| 11 – 13 Years old | 299 (26.7) | 348 (27.5) |  |
| 14 – 17 Years old | 482 (43.0) | 556 (44.0) |  |
| **Number of Complex Chronic Conditions (CCCs)** | | | |
| 1 CCC | 1021 (71.3) | 1120 (68.0) | 0.05 |
| 2 or More CCCs | 411 (28.7) | 528 (32.0) |  |
| **Hospital Encounters in 2020** | | | |
| 0 Hospital Encounters | 1177 (82.2) | 1279 (77.6) | 0.0016 |
| 1 or more Hospital Encounters | 255 (17.8) | 369 (22.4) |  |
