# Supplementary material for: Differences in COVID-19 testing perceptions among caregivers of children with medical complexity by rurality
Source: PLoS One. 2025 May 13;20(5):e0323651. doi: 10.1371/journal.pone.0323651 (PMC12074607; doi:10.1371/journal.pone.0323651)
Supplement: Attachment — Submitted filename: Response to Reviewers.docx [file pone.0323651.s003.docx]

PLOS One Submission Comments: Manuscript PONE-D-24-43670

*Differences in COVID-19 testing perceptions from caregivers of children with medical complexity by rurality*

| **Reviewer Comment** | **Author Response** | **Line Number** |
| --- | --- | --- |
| Reviewer #1 | | |
| Improve the abstract section and add a background section | Thank you for this feedback. We have expanded upon the background section of the abstract. | Lines 46-59 |
| Detail financial support received to fund the study | Thank you for noting this. Financial support information has been updated and is further detailed in the PLOS application. | Lines 27-30 |
| Relate the study to global commitment to universal health coverage and SDG3 | We agree with the importance of the global commitment to universal health coverage and UN SDG3 and have highlighted these principles in our discussion. | Lines 262-276 |
| Shed light on the impact of this study on future pandemic planning | We appreciate this insight. We have underscored the importance of this research field for pandemic planning in the study rationale and discussion. | Lines 93-99  Lines 258-276 |
| Detail the rationale of the study | Thank you for this suggestion. We have further defined the rationale of the study. | Lines 93-99 |
| Explain the validation process for translated questionnaire and training of interviewers | We appreciate the opportunity to discuss the validation process.  The NIH conducted a rigorous question development process. For our survey, we utilized the NIH’s required RADx-UP Common Data Elements in both English and Spanish (https://radx-up.org/research/cdes/). These questions underwent substantial community-involved development and were standardized/ required across the NIH’s RADx-UP programs.  For Spanish-language instruments, we employed a certified Spanish translator to review and translate all items. Additionally, a second Spanish translator at the University of Wisconsin Survey Center performed a back-translation and verification of all translated documents to ensure accuracy and consistency.  As the surveys were delivered by mail / internet, interviewers were not required for this study. | Lines 119-121 |
| Explain ethical consideration that had been accounted for vulnerable population | Thank you for highlighting this important topic. Research focused on studying how to improve care for children with complex illnesses is a national priority. While our research focuses on children with medical complexity, CMC were not the direct study subjects.  Additionally, socially vulnerable populations, including those who do not speak English, have disproportionately suffered from the pandemic, and research must include these subjects to understand and address their needs.  The study posed minimal risk to participants. The consent information sheet was written to be easily understood, and all study materials were translated into Spanish for non-English speaking subjects. Subjects were mailed a paper survey and could choose not to complete the survey. Subjects were not contacted in any other way, unless they gave permission for email contact for future survey waves. Minors were never contacted directly or asked to complete the survey, only adult parents or guardians were invited to participate in the study. | Lines 115-121 |
| What are other languages in Table 1 | Since participants reported over 30 primary household languages, we reported the most common languages (English and Spanish) and combined the others into a single category to facilitate analysis. Unfortunately, sample sizes were not sufficient to conduct a more detailed investigation. We have noted this in our limitations section | Lines 285-287 |
| Reviewer #2 | | |
| Introduction | | |
| The introduction section begins with this sentence: “COVID-19 testing is an invaluable tool in long-term pandemic management.[1]”  This way of in-text citation was used throughout the article. It must be noted that references are usually placed before punctuation (e.g., periods, commas, semicolons). Therefore, it would be prudent to look at reference placement in the entire manuscript and make any necessary adjustments. | Thank you for the suggestion. The formatting has been standardized throughout the manuscript. | Entire manuscript |
| This section is in need of a more compelling rationale that carries greater weight than the one presented here. | Thank you for this important comment. We have underscored the impact of COVID-19 testing for CMC throughout the introduction and further defined the rationale of the study. | Lines 71 - 99 |
| Methods | | |
| In the methods section, the authors write, “Survey content was derived from NIH’s RADx-UP Common Data Elements library[15]”  There should be a space between the reference and the text. And there should be a period after the reference. Please refer to the PLOS style guidelines and format your manuscript accordingly. | This sentence has been updated. Thank you for the detailed review. | Lines 105-106 |
| Additionally, transitions between paragraphs can be improved to make the reading smoother and more fluid. | Thank you for the suggestion. We have included transition phrases and separated larger sections of text into paragraphs to improve the flow. | Methods Section |
| Another important issue is the sampling technique. The authors have not mentioned what sampling technique they used. It is advisable to specify it for reasons of clarity and because of its impacts on the credibility of their findings. | Thank you for highlighting this topic. Our study utilized a convenience sampling method – all individuals who met inclusion criteria for the study were invited to participate. This has been defined in the methods section. | Lines 111-113 |
| Discussion | | |
| In this type of studies, the participants (i.e., those who respond to the survey) are more likely to be willing to take part in the study because they may be interested in the topic for a variety of reasons, or simply because they have extra time on their hands. I wonder if the authors have taken this aspect into consideration.  The authors state that “almost all reported wanting to know if their child had COVID-19, and rural caregivers were more likely than urban caregivers to state that their child’s health would be severely affected from COVID-19. This raises the question why rural populations would have lower testing intentions.” Could this observation be partly related to the nature of this study and the point raised in 3.1? | Thank you for your insightful comment. We acknowledge that participants who chose to respond to our survey might have a particular interest in the topic or more available time, which could influence their decision to participate.  To address this, we implemented several strategies to mitigate selection bias. We partnered with the University of Wisconsin survey center to conduct a mixed mode web and mail protocol designed to maximize response rates, data quality and representativeness of the pool of participants at each wave. The use of mailed surveys aimed to increase participation of lower income / lower education households over a web-only design. To maximize participation, several best practices were employed: dual language (English and Spanish) offerings, multiple contact attempts for each wave, and pre- and post-incentives ($5 pre and $50 post, contingent upon completion).  Lastly, we have included the frequency distributions (of variables available to us for both respondents and non-respondents) to ensure these differences are transparent for readers (S1 Table). To note, respondents and non-respondents exhibited similar distributions of rurality status and number of complex chronic conditions. The methods and results sections have been revised to incorporate the additional sensitivity analysis.  We believe these measures helped to reduce the impact of selection bias, although we acknowledge that it cannot be entirely eliminated. We have included a discussion of this limitation in our manuscript to provide a transparent account of our study's strengths and limitations. | Lines 159-161  Lines 175-177  Lines 281-283  Supplemental Table 1 |
