# Supplementary material for: Differences in COVID-19 testing perceptions among caregivers of children with medical complexity by rurality
Source: PLoS One. 2025 May 13;20(5):e0323651. doi: 10.1371/journal.pone.0323651 (PMC12074607; doi:10.1371/journal.pone.0323651)
Supplement: Attachment — Submitted filename: Response to Reviewers 3.4.25.docx [file pone.0323651.s004.docx]

PLOS One Submission Comments: Manuscript PONE-D-24-43670

*Differences in COVID-19 testing perceptions from caregivers of children with medical complexity by rurality*

| **Reviewer Comment** | **Author Response** | **Line Number** |
| --- | --- | --- |
| Editorial Comments | | |
| Please address the reviewer's concern by shortening the abstract section | Thank you for highlighting the abstract length. We have revised the abstract and it is currently 250 words. | Lines 46 - 69 |
| Replace "an academic medical center in the Midwest" in the methods section of the abstract with "an academic medical center in the Midwestern region of the United States" or other expressions indicating that this study was conducted in the United States. | Thank you for identifying the oversight. We have revised all geographic phrases in the manuscript to identify the country. | Line 54  Line 102  Line 274 |
| Please thoroughly copyedit the manuscript to identify and address typos ("decrease" was written as "decease" in the conclusion section of the abstract. | We appreciate your thorough review of the manuscript. We have carefully reviewed the manuscript to address any grammatical errors. | Entire manuscript |
